# Obituary

**Published:** 1852-10

**Authors:** 


### OBITUARY.

Of the many promising and eminent physicians of Philadelphia, whose premature deaths the profession has been called to mourn within the last few months, no one will be more generally or deeply lamented than Dr. Isaac Parrish, whose obituary we find in several of our exchanges. He died of dysentery, a short time since, after a few days' illness. The *Medical Examiner* truly remarks:

"Few men could be less spared than Dr. Parrish; he was essentially a working man in the profession, both in the daily routine of practical duties, and in everything calculated to advance and elevate it. To the pages of this journal, as well as of many of the other medical and daily papers, he was a constant and welcome contributor, and his articles were always characterized by sound judgment and practical bearing. He died in the prime of life, and in the full tide of a successful practice, beloved and respected by all who knew him."

Dr. Parrish impressed the stranger at once by a manner singularly frank and sincere, and the phrase "the just upright man," is one that now rises to our mind as descriptive of his character. In him we feel that our profession has lost an ornament.

